# Displacement of all three leaflets of tricuspid valve: A rare variant of Ebstein anomaly

**DOI:** 10.1002/ccr3.5555

**Published:** 2022-03-08

**Authors:** Zahra Khajali, Nahid Rezaeian, Zahra Ansari

**Affiliations:** ^1^ Rajaie Cardiovascular Medical and Research Center Iran University of Medical Sciences Tehran Iran

**Keywords:** anterior leaflet, Ebstein anomaly, rare variant, Tricuspid insufficiency

## Abstract

Ebstein anomaly, a rare congenital heart disease, is defined as displacement of hinge points of septal or posterior tricuspid leaflets but not anterior leaflet. Here, we report the case of a 35‐year‐old woman with an extremely rare pattern of Ebstein anomaly (EA) with all three tricuspid leaflets displaced downward to the apex.

## Graphical abstract

Ebstein anomaly is a rare congenital heart disease. EA is characterized by septal and posterior leaflets' downward displacement with the anterior leaflet remaining in the normal position. We present a rare case of EA with apical displacement of all three Tricuspid valve (TV) leaflets, in which TV replacement is recommended rather than repair.

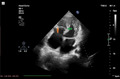

## 1 INTRODUCTION

Ebstein anomaly is a rare form of congenital heart disease with approximately one in 20,000 live births.^1^ Ebstein anomaly (EA) is characterized by septal and posterior leaflets' downward displacement into the body of right ventricle with the anterior leaflet remaining in the normal position.^2^, ^3^, ^4^ Here, we present a rare case of EA with apical displacement of all 3 leaflets of tricuspid valve.

## 2 PRESENTATION OF CASE

**FIGURE 1:**
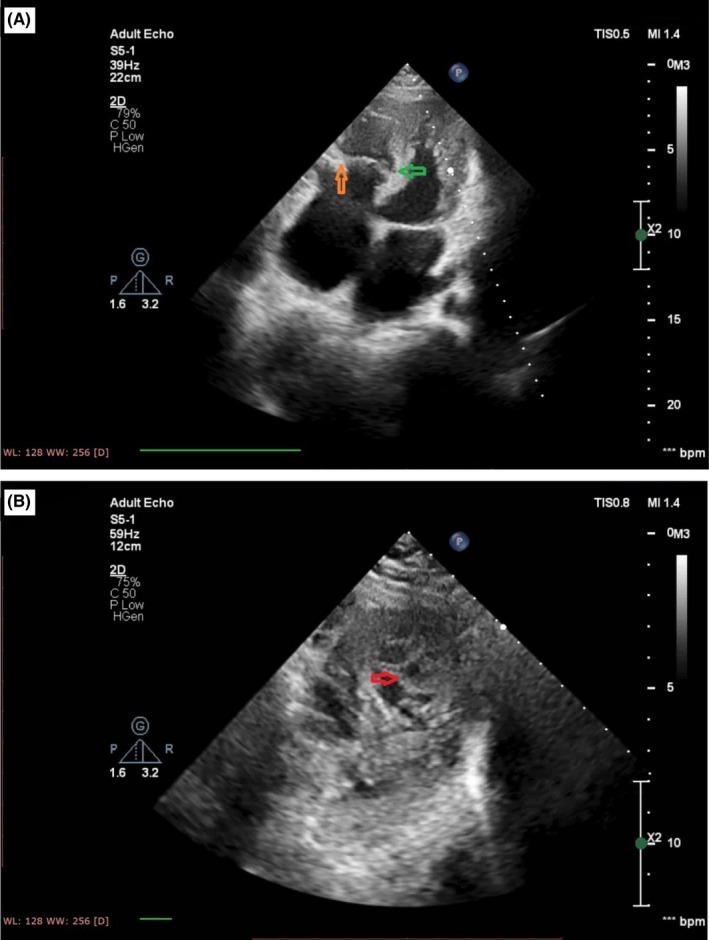
Transthoracic echocardiography. (A) Four‐chamber view showed apical displacement of all three tricuspid valve leaflets with no anterior leaflet elongation. (B) Parasternal short axis view showed apical non‐compaction of left ventricle as an associated anomaly

**FIGURE 2:**
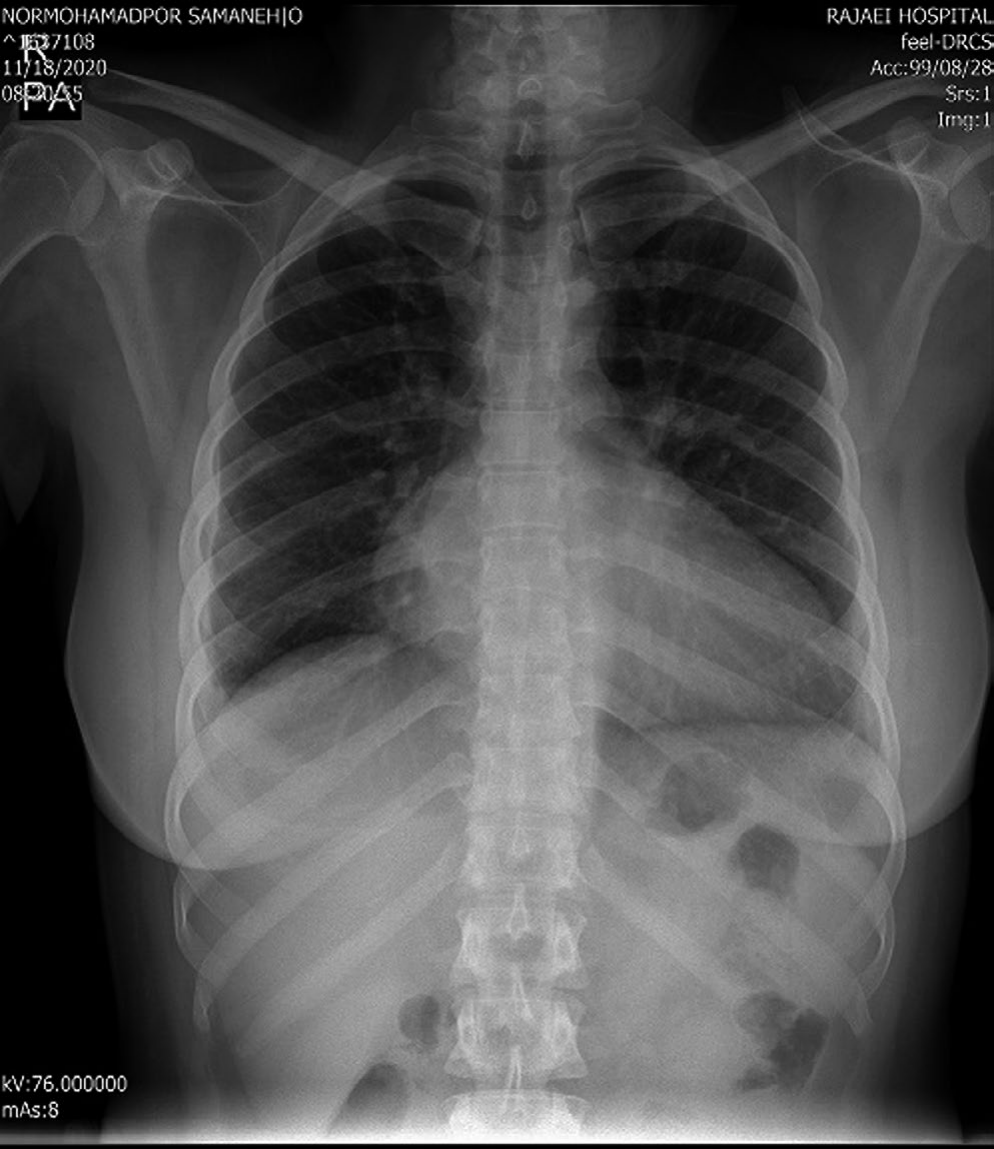
Anteroposterior chest X‐ray. Note increased cardiothoracic ratio due to enlarged right ventricle and also right atrium due to Ebstein anomaly and severe tricuspid regurgitation

**FIGURE 3:**
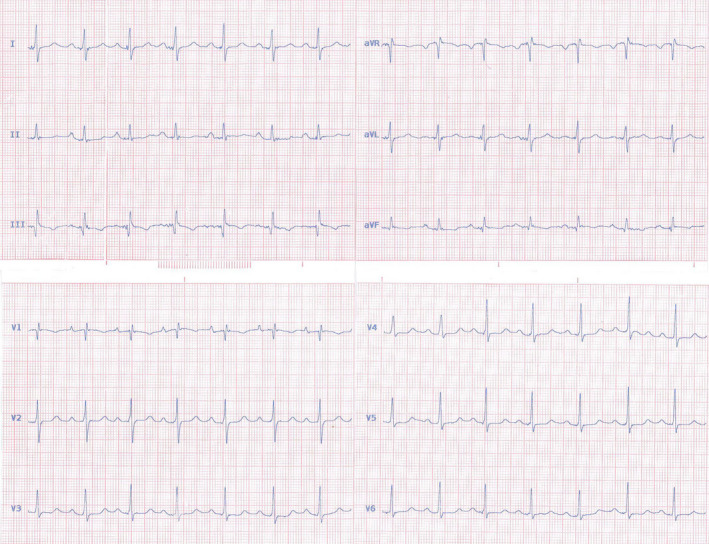
Electrocardiogram showed narrow QRS with first degree Aortic valve (AV) block. Note Q wave in II and a ventricular fibrillation (VF)

**FIGURE 4:**
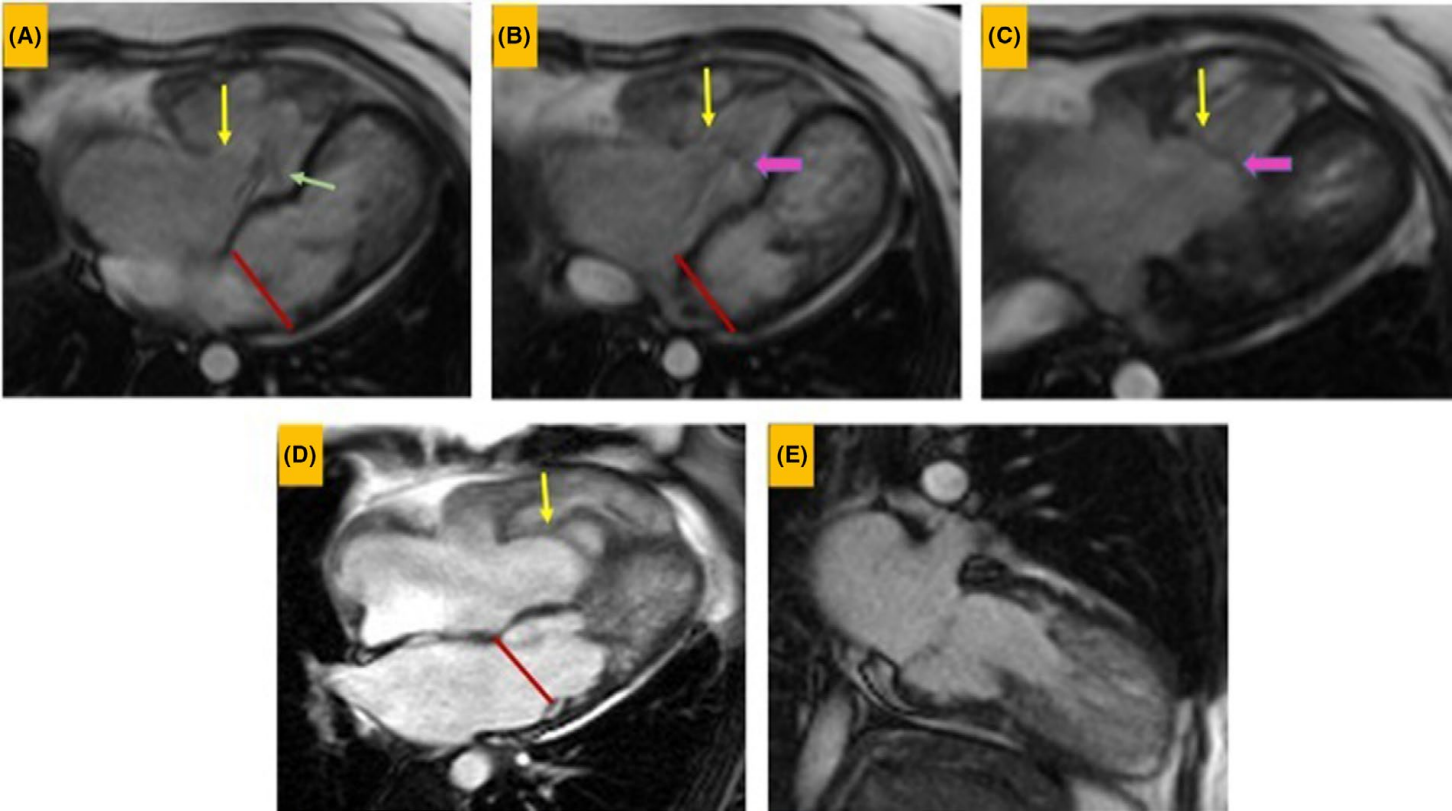
Cardiac magnetic resonance (CMR) image demonstrates apical displacement of all tricuspid valve leaflets. (A–D) show anterior tricuspid valve (yellow arrows), septal tricuspid (green arrow), and posterior tricuspid valve (thick pink arrows) displacement. The redline depicts the approximate level of the mitral valve. (E) Two‐chamber cine function shows non‐compaction left ventricle (LV)

A 35‐year‐old woman was referred to adult congenital heart disease department due to recently presented dyspnea on exertion and systolic murmur. Transthoracic echocardiography indicated an enlarged RA with apical displacement of septal (1.6 cm/m^2^), posterior (1.5 cm/m^2^), and also anterior (1.2 cm/m^2^) tricuspid leaflets (Figure 1). Severe right atrium enlargement and severe low gradient tricuspid regurgitation (TRG = 26 mmHg) were also recorded. Interatrial septum was aneurysmal with large PFO, but fortunately, no cyanosis was detected even in exercise. Left ventricular function was mildly reduced with hypertrabeculation and apical non‐compaction. Chest X‐ray showed increased cardiothoracic ratio more than 50% with enlarged RA size (Figure 2). Electrocardiogram showed normal sinus rhythm with first degree Aortic valve (AV) block, Q in leads III and a ventricular fibrillation (VF) and narrow QRS (Figure 3). 48‐h rhythm holter monitoring was done also due to palpitation, but no arrhythmia was detected. Sinus tachycardia was the only finding in the rhythm holter monitoring. Cardiac magnetic resonance (CMR) was done to evaluate cardiac function, anatomy, and fibrosis. It has demonstrated an extraordinary case of Ebstein anomaly with apical displacement of all anterior (35 mm/m^2^ BSA), septal (18 mm/m^2^ BSA), and posterior (20 mm/m^2^ BSA) tricuspid valve leaflets (Figure 4). Anterior tricuspid valve leaflet was short and had some fenestration with severe TR. CMR also mentioned a prominent ridge in RA at the site of AV groove, which protruded to RV cavity with attachment of anterior Tricuspid valve (TV) leaflet to it. Medical treatment with beta‐blocker and low dose duretic begun due to dyspnea and palpitation, and surgical consult was done. The patient was a candidate for valvular surgery, but the patient did not accept it. In medical follow‐ups, the patient signs and symptoms were improved although surgery is still the treatment of choice for our patient.

## 3 DISCUSSION

Ebstein anomaly is essentially a tricuspid valve disorder which begins in the embryonic stage by failure of delamination.^2^, ^5^ Due to failure in delamination of inner layer of inlet part of right ventricle in EA, we see adherence of septal and posterior leaflets to the underlying myocardium and as a consequence, we saw downward displacement of hinge points of septal and posterior leaflets from atrioventricular junction.^2^, ^5^, ^6^ In contrast to septal and posterior leaflets, anterior leaflet usually remains in the normal position due to different embryonic origin of anterior leaflet versus posterior and septal leaflets.^4^, ^7^ Septal (medial) and posterior (inferior) leaflets originate from the tricuspid gully,^7^ but the anterior leaflet originates from the lateral endocardial cushion and lateral conus,^2^ so that, anterior tricuspid leaflet is very rarely affected in EA and the attach point of the septal and inferior leaflets never apically displaced beyond the junction between ventricular inlet and apicotrabecular component of RV,^1^, ^7^ and the junctional hinge of the anterior leaflet is very rarely affected.^2^, ^7^

In our case, in which the symptoms represented in adulthood, a rare variant of EA was seen with downward displacement of all three tricuspid leaflets. Having enough information about the valve anatomy, especially rare variants is so important because the surgical plan may be different in special cases. As in case of anterior leaflet displacement in Ebstein cases, mostly replacement is needed rather than valvar repair.^8^ So, if an EA is suspected, the physician should be aware of rare conditions and look for them carefully. Ebstein cases may represent at different ages due to severity of structural and functional abnormality and also presence of associated defects,^9^ such as our patient who well‐tolerated till 35 years old and passed two pregnancies with no complaints of dyspnea or right side failure symptoms. Apical disposition of all three tricuspid leaflets is rarely reported in literature. There was reported few cases of isolated anterior leaflet displacement and not septal and posterior ones, and displacement of septal or posterior leaflets is the regular kind of EA, but apical downward of all three ones was not reported till now. Having enough knowledge about rare variations will help the surgeon to the best decision making for the patient.

## CONFLICT OF INTEREST

The authors have no conflicts of interest to declare.

## AUTHOR CONTRIBUTIONS

ZK contributed to the conception and design of the study and involved in the provision of study materials of patients, data analysis and interpretation, and manuscript writing. ZK, NR, and ZA contributed to radiological evaluations. NR and ZA contributed to data analysis and interpretation. All authors participated in the collection and/or assembly of data. All authors read, revised, and approved the final manuscript.

## CONSENT

Written informed consent was obtained from the patient to publish this report in accordance with journal's patient consent policy.

## Data Availability

Raw data supporting the findings of this study are available from the corresponding author [Z.A.] on request.
